# Identification of Key Functional Genes and LncRNAs Influencing Muscle Growth and Development in Leizhou Black Goats

**DOI:** 10.3390/genes14040881

**Published:** 2023-04-08

**Authors:** Xiuhui Zhao, Junning Ye, Xunkai Lin, Huiwen Xue, Xian Zou, Guangbin Liu, Ming Deng, Baoli Sun, Yongqing Guo, Dewu Liu, Yaokun Li

**Affiliations:** 1 College of Animal Science, South China Agricultural University, Guangzhou 510642, Chinadwliu@scau.edu.cn (D.L.); 2 State Key Laboratory of Livestock and Poultry Breeding, Guangdong Key Laboratory of Animal Breeding and Nutrition, Institute of Animal Science, Guangdong Academy of Agricultural Sciences, Guangzhou 510640, China; 3 National Local Joint Engineering Research Center of Livestock and Poultry, South China Agricultural University, Guangzhou 510642, China

**Keywords:** Leizhou black goat, lncRNA, longissimus dorsi, meat quality, meat yield, mRNA, protein methylation, skeletal muscle

## Abstract

Meat yield and quality are important economic traits of livestock. Herein, longissimus dorsi (LD) muscles of Leizhou black goats aged 0, 3, and 6 months were used to identify differentially expressed messenger RNAs (mRNAs) and long non-coding RNAs (lncRNAs) by high-throughput RNA sequencing. Gene Ontology (GO) and Kyoto Encyclopedia of Genes and Genomes (KEGG) analyses were used to analyze differentially expressed genes. Expression levels of regulator of calcineurin 1 (*RCAN1*) and olfactory receptor 2AP1 (*OR2AP1*) were significantly different in LD muscles of goats aged 0, 3, and 6 months, indicating potentially important roles in postnatal muscle development. Differentially expressed lncRNAs and mRNAs were mainly enriched in biological processes and pathways related to cellular energy metabolism, consistent with previous studies. Three lncRNAs, TCONS_00074191, TCONS_00074190, and TCONS_00078361, may play a cis-acting role with methyltransferase-like 11B (*METTL11B*) genes and participate in the methylation of goat muscle proteins. Some of the identified genes may provide valuable resources for future studies on postnatal meat development in goat muscles.

## 1. Introduction

Skeletal muscle mass accounts for 40–60% of the body weight of mammals. It is economically important and a major focus in breeding goats with improved traits. The main functions of skeletal muscles are movement and protection, and they are also responsible for regulating the structure and metabolism of the body [1]. Skeletal muscle development in domestic animals can be divided into prenatal and postnatal stages; the number of muscle fibers increases before birth and the volume of muscle fibers increases after birth. In the embryonic stage, mesenchymal stem cells of the mesoderm differentiate into mononuclear myoblasts, then into spindle multinuclear myotubes, and finally into myofibers [2]. In later development, muscle fibers gradually differentiate into slow or fast types to form a complete skeletal muscle [3].

Long non-coding RNAs (lncRNAs) are ncRNAs >200 bp in length, with low or no protein-coding potential. Although lncRNA sequences are poorly conserved, studies have shown that a large number of lncRNAs with similar functions have short conserved elements [4]. Based on the genomic location relative to nearby protein-coding genes, lncRNAs can be divided into sense lncRNAs, anti-sense lncRNAs (lncNATs), intergenic lncRNA (lincRNAs), intronic lncRNAs, and bidirectional lncRNAs [5]. These RNAs usually regulate epigenetic silencing via chromatin remodeling. They also regulate splicing, recruit transcription factors, and regulate the stability of mRNAs. LncRNAs are present in many organisms, and they influence numerous physiological processes including ontogeny and disease occurrence [6,7,8,9,10].

In recent years, many studies have confirmed that ncRNAs are also important members of the muscle regulatory network. Metastasis-associated lung adenocarcinoma transcript 1 (*Malat1*) expression is increased during differentiation of myoblasts into myotubes, and proliferation of myoblasts was inhibited upon targeted knockdown of *Malat1* using a small interfering RNA (siRNA). This reveals that *Malat1* is a novel downstream target of myostatin, and it regulates myogenesis [11]. The expression of Linc-RNA activator of myogenesis (*Linc-RAM*) was upregulated during myogenesis, while muscle regeneration of *Linc-RAM* knockout mice was impaired. *Linc-RAM* can directly bind MyoD to regulate the expression of myogenic genes and facilitate assembly of the MyoD–Baf60c–Brg1 complex, thereby promoting myogenesis [12]. Staufen-mediated mRNA decay (SMD) occurs in mouse cells through partially complementary mRNA-lncRNA base pairing, and it is triggered by mouse half-STAU1-binding site (1/2-sbsRNA) to regulate myogenesis of C2C12 cells [13]. Overexpression of sirtuin 1 (*Sirt1*) antisense (AS) lncRNA promotes myoblast proliferation but inhibits differentiation. *Sirt1* AS lncRNA interacts with the Sirt1 3′ untranslated region (UTR), prolonging the half-life of Sirt1 mRNA, and promoting Sirt1 translation and inhibiting muscle formation by competing with miR34a [14].

Xu et al. (2012) identified 185 unigenes from longissimus dorsi (LD) muscles of Hainan black sheep aged 2, 6, 12, and 24 months, most of which were differentially expressed genes (DEGs) involved in energy metabolism and muscle contraction [15]. The first reported systematic identification of lncRNAs from RNA sequencing (RNA-seq) data during goat skeletal muscle development was published by Zhan et al. (2016) who identified 577 differentially expressed lncRNAs from fetal (45, 60, and 105 days of gestation) and postnatal (3 days after birth) goat LD muscles [16]. Ling et al. (2019) identified 547 differentially expressed lncRNAs during skeletal muscle development in domestic goats (Capra hircus), which included seven fetal stages at 45 (F45), 65 (F65), 90 (F90), 120 (F120), and 135 (F135) days, and 24 h (B1) and 90 (B90) days after birth [17].

In the present study, high-throughput sequencing was performed on LD muscles of Leizhou black goats aged 0, 3, and 6 months; differentially expressed lncRNAs and mRNAs were identified, and their temporal expression characteristics and potential functions were investigated. The results provide insight into the functions of lncRNAs and mRNAs in goat skeletal muscle development.

## 2. Materials and Methods

### 2.1. Animals and Sample Collection

Nine Leizhou black goats were divided equally into three groups according to age (0, 3, or 6 months). All animals were raised under the same conditions with free access to food and water under natural lighting. All animals were slaughtered in accordance with animal welfare procedures, and after slaughter, LD muscle samples were collected at the three growth stages (M0, M3, and M6). Tissue samples were immediately frozen in liquid nitrogen and stored at −80 °C until analysis. All experimental procedures and sample collection methods complied with the Regulation on the Administration of Laboratory Animals (CLI.2.293192, 2017 Revision, The State Council of China) and were performed in strict accordance with the guidelines of the Institutional Animal Care and Use Committees of South China Agricultural University (approval No. 2018-P002).

### 2.2. RNA Isolation, Library Preparation, and Sequencing

Total RNA was extracted using TRIzol reagent (Invitrogen, Carlsbad, CA, USA) according to the manufacturer’s instructions. RNA degradation and contamination were monitored on 1% agarose gels. RNA purity was checked using a NanoPhotometer spectrophotometer (IMPLEN, CA, USA). RNA integrity was assessed using an RNA Nano 6000 Assay Kit and a Bioanalyzer 2100 system (Agilent Technologies, CA, USA).

For each sample, 1 µg of RNA was used as input material, sequencing libraries were generated using an NEBNext Ultra RNA Library Prep Kit for Illumina (NEB, USA) following manufacturer’s recommendations, and index codes were added to attribute sequences to each sample. Briefly, mRNA was purified from total RNA using poly-T oligo-attached magnetic beads. Fragmentation was carried out using divalent cations under elevated temperatures in NEBNext First Strand Synthesis Reaction Buffer (5×). First-strand cDNA was synthesized using random hexamer primer and M-MuLV Reverse Transcriptase (RNase H-). Second-strand cDNA synthesis was subsequently performed using DNA Polymerase I and RNase H. Remaining overhangs were converted into blunt ends via exonuclease/polymerase activities. After adenylation of 3′ ends of DNA fragments, NEBNext adaptors with a hairpin loop structure were ligated to prepare for hybridization. In order to select cDNA fragments 250–300 bp in length, the library fragments were purified using an AMPure XP system (Beckman Coulter, Beverly, USA). Next, 3 µL of USER Enzyme (NEB) was incubated with size-selected, adaptor-ligated cDNA at 37 °C for 15 min, followed by 5 min at 95 °C before PCR. Amplification was then performed using Phusion High-Fidelity DNA Polymerase, Universal PCR Primers, and Index (X) Primer. Finally, the PCR products were purified using an AMPure XP system, and library quality was assessed on an Agilent Bioanalyzer 2100 system.

Clustering of index-coded samples was performed on a cBot Cluster Generation System using a TruSeq PE Cluster Kit v3-cBot-HS (Illumina) according to the manufacturer’s instructions. After cluster generation, library preparations were sequenced on an Illumina Novaseq platform, and 150 bp paired-end reads were generated.

### 2.3. Data Analysis

Raw reads in fastq format were first processed through NGQC Perl scripts. To obtain high-quality filtered reads, we removed (1) reads with adapters (NGQC software), (2) reads with an N proportion > 0.001 (where N is an unknown base), and (3) low-quality reads (reads with Qphred ≤ 20 bases accounting for >50% of the entire read length). The Q20, Q30, and GC content values of the clean data were calculated, and all downstream analyses were performed on the clean, high-quality data.

Reference genome and gene model annotation files were downloaded from genome websites. The index of the reference genome was built using Hisat2 v2.0.5 and paired-end clean reads were aligned to the reference genome using Hisat2 v2.0.5. We selected Hisat2 as the mapping tool because it can generate a database of splice junctions based on the gene model annotation file, and thus generate better mapping results than other non-splice mapping tools. The mapped reads for each sample were assembled by StringTie (v1.3.3b) using a reference-based approach [18]. StringTie uses a novel network flow algorithm as well as an optional de novo assembly step to assemble and quantitate full-length transcripts representing multiple splice variants for each gene locus.

FeatureCounts v1.5.0-p3 was used to count the number of reads mapped to each gene, and the fragments per kilobase of exon model per million mapped fragments (FPKM) value of each gene was calculated based on the length of the gene and the number of reads mapped to the gene.

### 2.4. LncRNA Identification

Before screening, Cu merge was used to create a set of transcripts. LncRNA screening was carried using the following steps:

Step 1: select transcripts with two exons;

Step 2: from the results of step 1, select transcripts with a length > 200 bp;

Step 3: annotate the above transcripts using Cu compare software;

Step 4: calculate the expression level of each transcript using Cu quant and select transcripts with FPKM 0.1;

Step 5: coding of potential transcripts using Coding-Non-Coding-Index (CNCI) (v2) [19], Coding Potential Calculator (CPC) 0.9-r2 [20], and Pfam Scan v1.3 [21,22]. The intersections of transcripts without coding potential based on the above three software packages using default parameters were predicted as the lncRNA dataset [23].

### 2.5. Expression Analysis

Differential expression analysis of two groups was performed using the DESeq R package (1.8.3), and *p*-values were adjusted using the Benjamini and Hochberg method. A corrected *p*-value of 0.05 was set as the threshold for significantly differential expression by default.

### 2.6. Gene Ontology (GO) and Kyoto Encyclopedia of Genes and Genomes (KEGG) Enrichment Analyses

In this study, mRNAs within a 100 kb window upstream or downstream of differentially expressed genes served as a cis-target mRNA dataset of differentially expressed lncRNAs (DE-lncRNAs). GO enrichment analysis was used on target gene candidates of DE-mRNAs and DE-lncRNAs. GO-seq based on Wallenius non-central hyper-geometric distribution [24], which can adjust for gene length bias, was implemented for GO enrichment analysis.

KEGG [25] is a database resource for understanding high-level functions of biological systems such as cells, organisms, and ecosystems from molecular-level information, especially large-scale molecular datasets generated by genome sequencing and other high-throughput experimental technologies [http://www.genome.jp/kegg/ (accessed on 1 July 2022)]. We used KOBAS [26] software to test the statistical enrichment of target gene candidates in KEGG pathways.

### 2.7. Target Gene Prediction

A previous study showed that lncRNAs play a cis-regulatory role on their colocalized genes [27]. Colocalization is the action of lncRNAs on adjacent target genes [17]. For each lncRNA locus, the 100 kb upstream and downstream regions were chosen to screen co-located genes using UCSC Genome Browser. DE-lncRNAs and their corresponding DE-mRNAs in each age group were screened to predict the main functions of the lncRNAs.

### 2.8. qRT-PCR Verification

The cDNAs for quantitative real-time polymerase chain reaction (qRT-PCR) were synthesized using a PrimeScript RT Reagent Kit With gDNA Eraser (TaKaRa, Dalian, China) and qRT-PCR was performed using 2 × Ultra SYBR Green qPCR Mix (Cistro, Shanghai, China). The 20 μL reactions contained 10 μL Mix, 1 μL forward and reverse primers, 2 μL cDNA, and 6 μL water. Reactions were incubated in a 96-well optical plate at 95 °C for 10 min, followed by 40 cycles of 5 s at 95 °C and 20 s at 60 °C. Each sample was run in triplicate for analysis. Goat β-actin served as the endogenous control for mRNA and lncRNA expression analyses. After the PCR cycles, we performed melting curve analysis to confirm specific generation of the expected PCR product. Primer sequences are listed in Table 1.

## 3. Results

### 3.1. Sequencing Data Quality Control

Raw reads from the 0-, 3-, and 6-month-old groups were analyzed for quality control before further analyses (Table 2). The Q30 value for each sample exceeded 91%. The mapped ratio of clean reads to the reference genome was >82%, and >80% of sequences were uniquely mapped to the genome of C. hircus, indicating that the sequencing data was of high quality and suitable for subsequent analyses.

**Figure 1:**
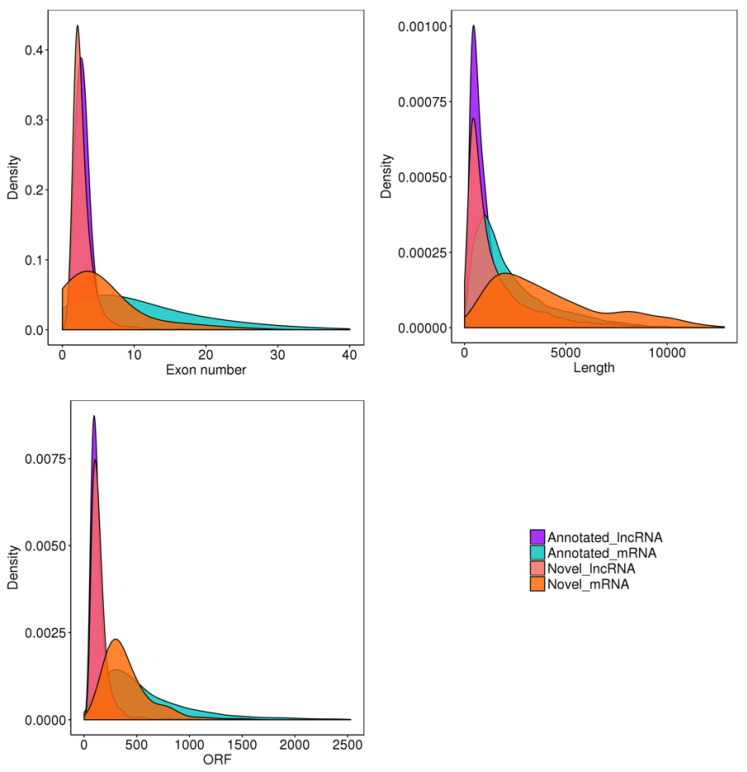
Goat skeletal muscle structure and conservation. ORF stands for open reading frame.

Structural characteristics are important for understanding lncRNAs and mRNAs. Our results showed that lncRNAs had fewer exons than mRNAs, they were shorter in length, and their open reading frames (ORFs) were also shorter than those of mRNAs (Figure 1).

### 3.2. Differential Expression of mRNAs and LncRNAs

To investigate the key mRNAs and lncRNAs involved in regulating goat skeletal muscle development, we used RNA-seq datasets from three timepoints to characterize their time-specific expression patterns (Table 3). When comparing DE-mRNAs across the three developmental stages, we found 192 DE-mRNAs (103 upregulated) between M0 and M3, 288 DE-mRNAs (167 upregulated) between M0 and M6, and 159 DE-mRNAs (65 upregulated) between M6 and M3. When analyzing these DE-mRNAs, we found that 59, 134, and 58 mRNAs were uniquely expressed in one of the two samples in M0 vs. M3, M0 vs. M6, and M6 vs. M3 comparisons, respectively. We also analyzed DE-lncRNAs between M0 and M3, M0 and M6, and M6 and M3, and detected 55 (23 upregulated), 83 (45 upregulated), and 33 (10 upregulated) DE-lncRNAs. We found that 28, 59, and 18 lncRNAs were only expressed in the M0 and M3, M0 and M6, and M6 and M3 comparisons, respectively.

### 3.3. GO Analyses of DE-mRNAs and Target Genes of DE-lncRNAs

In the M0 vs. M3 comparison, the top GO terms that were significantly related to the DE-mRNAs included respiratory chain and generation of precursor metabolites. In the M0 vs. M6 comparison, the most significantly enriched GO terms for the DE-mRNAs were associated with ribosome, structural constituent of ribosome, ribonucleoprotein complex, translation, structural molecule activity, and non-membrane-bounded organelle. In the M6 vs. M3 comparison, the top processes for down- and upregulated mRNAs were associated with urea transmembrane transporter activity, urea transport, one-carbon compound transport, and urea transmembrane transport (Table 4).

In the M0 vs. M3 and M0 vs. M6 comparisons the DE-lncRNA target genes, there were no GO terms with a *p*-value < 0.05. In the M6 vs. M3 comparison, the top processes for down- and upregulated DE-lncRNA target genes were associated with urea transmembrane transporter activity, urea transport, one-carbon compound transport, and urea transmembrane transport (Table 5).

### 3.4. KEGG Analyses of DE-mRNAs and Target Genes of DE-lncRNAs

KEGG pathway analysis was applied to identify pathways enriched in DE-mRNAs and target genes of DE-lncRNAs. The most significantly enriched pathways of DE-mRNAs were detected in the M0 and M6 comparison (ribosome, biosynthesis of amino acids, and glycolysis/gluconeogenesis; Table 6). There were no KEGG pathways with a *p*-value < 0.05 detected in the M0 vs. M3 and M6 vs. M3 comparisons. The target genes of DE-lncRNAs (Table 7) were linked to 2-oxocarboxylic acid metabolism (M0 vs. M3) and ubiquitin-mediated proteolysis (M0 vs. M6).

### 3.5. Predicted Target Genes of DE-lncRNAs

There were only three biological replicates in our sample, which was insufficient for co-expression analysis. We therefore intersected DE-mRNAs and genes co-located with DE-lncRNAs, and 13 pairs of genes with possible interactions were obtained that may provide insight into mRNA–lncRNA interactions in skeletal muscle development (Table 8). Notably, expression of *METTL11B* was downregulated continuously in all three stages.

### 3.6. Validation of RNA-Seq Data

**Figure 2:**
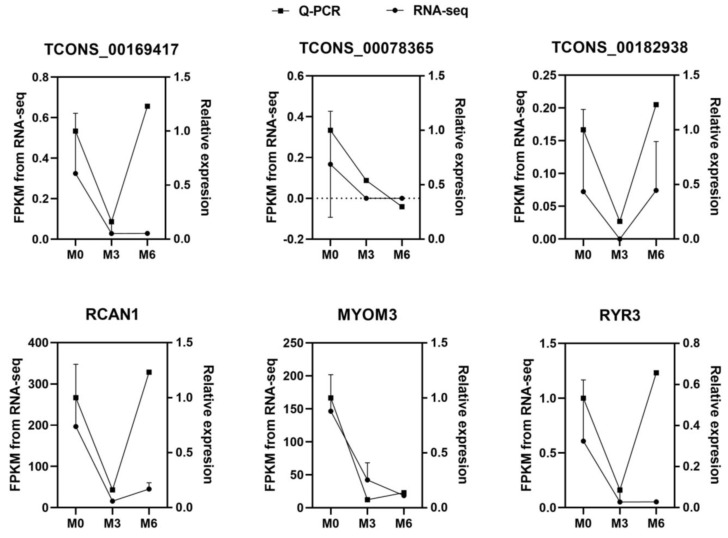
qRT-PCR results for mRNAs and lncRNAs and FPKM from RNA-seq of M0, M3, and M6 groups.

To validate the reliability of the RNA-seq results, three DE-lncRNAs (TCONS_00169417, TCONS_00078365, and TCONS_00182938) and three DE-mRNAs (*RCAN1*, *MYOM3*, and *RYR3*) were selected for qRT-PCR analysis (Figure 2). The expression patterns of the DE-lncRNAs and DE-mRNAs were found to be consistent with those of the RNA-seq results, which confirmed the reliability of the sequencing results.

## 4. Discussion

In this study, we systematically explored lncRNAs and mRNAs in LD muscle in goat during three developmental stages. We found that expression of lncRNAs and mRNAs was stage-specific. We can better understand lncRNAs and mRNAs by combining multiple structural features. Consistent with previous research, lncRNAs tended to contain fewer exons than protein-coding transcripts, and they were shorter than mRNAs due to having fewer exons. In addition, lncRNAs in the datasets also had shorter ORFs than mRNAs [28]. Additionally, we performed qRT-PCR verification of the differentially expressed genes obtained by sequencing, which validated the time-specific lncRNA and mRNA expression patterns, and the accuracy of gene expression quantification.

The regulation of calcineurin (RCAN), an endogenous calcineurin (CN) inhibitory protein, and RCAN1 has been widely studied. *RCAN1* plays an important role in many signaling pathways. It inhibits a series of downstream signaling events by inhibiting the calcineurin-nuclear factor of activated T cell (CN-NFAT) signaling pathway. *RCAN1* participates in the regulation of superoxide dismutase 1 (*SOD1*) by stimulating *SOD1* expression and improving SOD1 enzyme activity [29]. RCAN1 can also enhance the stability of LκBα protein, which forms a complex with NF-κB and inhibits the NF-κB signaling pathway [30]. Through analysis of DE-mRNAs and DE-lncRNAs, we found that expression of RCAN1 was significantly different in the three stages, and was highest at M0. Therefore, we speculate that regulation of CN plays an important role in muscle growth and development in goats after birth.

In the present study, we found that expression of *OR2AP1* was significantly different in the three stages, and it increased as the growth of goats progressed. There has been little research on *OR2AP1*, so we looked for a link between *OR2AP1* and muscle development in its family of genes. *OR* is mainly expressed on the neuronal surface of the olfactory epithelium, but studies have found that it is also expressed in primitive embryonic red blood cells [31], heart, testis, and prostate of male animals, etc. [32]. For example, the expression of the *OR* gene in sperm plays an important role in flagellar vitality and sperm tropism [33]. It has been shown that *mOR23* is necessary for normal skeletal muscle regeneration because the absence of *mOR23* leads to increased branching of muscle fibers, often resulting in muscular dystrophy [34]. Overexpression of *mOR23* in the muscles resulted in decreased muscle fiber branching after muscle regeneration in non-malnourished mice and decreased the severity of muscle fiber branching in the muscle of malnourished mice [35]. It has also been shown that AzA relies on *Olfr544* to induce skeletal muscle mitochondrial biogenesis [36]. Therefore, we reasonably speculated that *OR2AP1* plays an important role in the normal development of goat muscle.

Skeletal muscle requires a great deal of energy and proteins to develop [15]. It has been reported that skeletal muscle hypertrophy requires an increase in the rate of protein synthesis, and one way in which this can be achieved is by increasing the translational capacity of muscle through ribosome biogenesis [37]. In the M0 and M6 comparison, five of the top 20 enriched GO terms were related to ribosomes, consistent with this prediction.

Goats are ruminants, and the urea nitrogen cycle is very important for maintaining nitrogen balance [38]. There are two main ways for urea to enter the rumen: from saliva and directly from the blood through the rumen wall epithelium. Due to the low permeability of the cell membrane to urea, effective urea capture must be mediated by transporters. At present, the main transporters mediating urea transport in the rumen epithelium are believed to be the UT-B channel protein and some aquaglyceroporins. Gastrointestinal microorganisms can express and secrete the urea-decomposing enzymes that are lacking in mammals, and they can decompose urea into ammonia as a nitrogen source for their growth and reproduction. Furthermore, microbial products such as vitamins, SCFAs, peptides, and microorganisms themselves (high-quality proteins) can be absorbed and utilized by host animals. In this process, urea nitrogen is preserved and reused, and converted into a variety of nutritional molecules for use by the host animals [39,40,41]. In our study, numerous DE-mRNAs and DE-lncRNAs in the M6 vs. M3 comparison were enriched in pathways related to urea, indicating that rumen urea transporters start to act between 3 months and 6 months of age, which has an important effect on the development of skeletal muscle.

*METTL21C* has protein lysine N-methyltransferase activity and is highly expressed in mouse skeletal muscle [42]. Knockdown of the *METTL21C* gene in mouse skeletal muscle decreases the trimethylation level of valine casein protein (VCP/p97), increases the accumulation of autophagic vacuoles, and lowers skeletal muscle endurance [43]. *METTL21C* methylation modifies heat shock protein family A member 8 (*HSPA8*) protein, resulting in decreased expression of myocyte enhancer factor 2D (*MEF2D*) in mouse skeletal muscle [44]. In our study, expression of *METTL11B* decreased continuously in all three stages, and three lncRNAs co-localized with *METTL11B*, among which expression levels of TCONS_00074191 and TCONS_00074190 were increased from M0 to M3, and expression levels of TCONS_00078361 were increased from M0 to M6. Therefore, we speculate that the interactions of TCONS_00074191, TCONS_00074190, and TCONS_00078361 with *METTL11B* affect the development of goat muscle. *NBEA* regulates neuronal membrane protein transport and is essential for central and neuromuscular synaptic development and function [45,46]. *NBEA* is a candidate gene in chicken and pig skeletal muscle development [46]. In our study, the expression level of *NBEA* in the skeletal muscle of Leizhou black goats increased significantly from 0 months to 6 months of age, and two lncRNAs, TCONS_00045045 and TCONS_00045031, are co-located with them. We speculated that *NBEA* might be related to the development of longissimus dorsi muscles in goats.

## 5. Conclusions

In conclusion, we found that expression levels of *RCAN1* and *OR2AP1* were significantly different in LD muscle of goats aged 0, 3, and 6 months, suggesting that these proteins may play important roles in postnatal muscle development. Our results showed that DE-lncRNAs and DE-mRNAs were mainly enriched in biological processes and pathways related to cellular energy metabolism, consistent with previous studies. The three lncRNAs TCONS_00074191, TCONS_00074190, and TCONS_00078361 may play a cis-acting role with *METTL11B*, and participate in the methylation of goat muscle proteins. Some of the identified genes may provide valuable resources for future studies on postnatal meat development in goat muscles.

## Figures and Tables

**Table 1:** Sequences of primers used in qRT-PCR.

Gene	Primer Sequence 5′→3′	Product Length (bp)
TCONS_00169417	CCCGATTCCCCAGATAGCGA	117
	ACCGGATAAAGATCGGCTCG	
TCONS_00078365	AGGCAGGAATTGCGGTGTAT	288
	CCCAGGATCAGTCAACACCA	
TCONS_00182938	TTTCTTCACTGCCATCCTCCC	250
	AAGTTCTGTTGCCTTCCCCG	
*RCAN1*	GTTTGTATGTAGAGTTGCG	437
	TTGATGTATTAGTGGGGGT	
*MYOM3*	CACATTCTTCTCCCGGTCCC	124
	TGCGAGAGCAAAAACAGAAGC	
*RYR3*	TGGTCATCAACACGCCATCT	280
	ATGGTTGTGTACCAGGCGAG	

**Table 2:** Quality statistics for sequencing data.

Sample Name	Raw Reads	Clean Reads	Q20	Q30	Total Mapped	Multiple Mapped	Uniquely Mapped
M0_1	91,451,366	90,296,570	97.0%	92.1%	81,031,701 (94.2%)	11,681,824 (12.9%)	71,341,877 (81.2%)
M0_2	99,145,950	97,674,536	97.3%	92.6%	91,661,001 (95.9%)	1,491,508 (9.7%)	81,171,493 (86.2%)
M0_3	97,522,592	96,325,710	97.0%	91.9%	91,851,053 (96.4%)	1,741,407 (7.0%)	81,111,646 (89.4%)
M3_1	93,972,708	92,817,732	96.8%	91.5%	81,171,540 (92.8%)	11,311,379 (11.1%)	71,861,161 (81.7%)
M3_2	115,506,982	114,030,402	97.5%	93.4%	101,861,223 (91.1%)	11,141,916 (10.6%)	91,721,307 (80.4%)
M3_3	109,977,218	108,672,712	97.4%	93.1%	101,661,879 (92.6%)	1,921,360 (9.1%)	91,731,519 (83.5%)
M6_1	91,389,622	90,146,316	97.5%	93.4%	81,141,246 (94.4%)	1,011,263 (7.8%)	71,131,983 (86.7%)
M6_2	109,277,192	107,969,018	97.4%	93.1%	101,741,280 (93.3%)	11,131,444 (11.2%)	81,601,836 (82.1%)
M6_3	115,475,130	114,090,040	97.5%	93.4%	101,911,408 (93.7%)	11,511,743 (11.8%)	91,401,665 (81.9%)

**Table 3:** Number of DE-mRNAs and DE-lncRNAs at different developmental stages.

		Upregulated	Downregulated	Uniquely Expressed	Total
DE-mRNAs	M0 vs. M3	103	89	59	192
	M0 vs. M6	167	121	134	288
	M6 vs. M3	65	94	58	159
DE-lncRNAs	M0 vs. M3	23	32	28	55
	M0 vs. M6	45	38	59	83
	M6 vs. M3	10	23	18	33

**Table 4:** The most enriched GO terms of DE-mRNAs.

GO Accession	Description	Term Type	*p*-Value	Gene Count
**M0 vs. M3**				
GO:0070469	respiratory chain	CC	3.23 × 10^−3^	6
GO:0006091	generation of precursor metabolites and energy	BP	7.48 × 10^−3^	10
**M0 vs. M6**				
GO:0005840	ribosome	CC	4.65 × 10^−27^	59
GO:0003735	structural constituent of ribosome	MF	1.08 × 10^−26^	58
GO:0030529	ribonucleoprotein complex	CC	1.86 × 10^−23^	64
GO:0006412	translation	BP	9.49 × 10^−22^	67
GO:0005198	structural molecule activity	MF	4.10 × 10^−20^	73
GO:0043228	non-membrane-bound organelle	CC	1.54 × 10^−15^	106
GO:0043232	intracellular non-membrane-bound organelle	CC	1.54 × 10^−15^	106
GO:0044444	cytoplasmic part	CC	5.13 × 10^−10^	114
GO:0044391	ribosomal subunit	CC	5.25 × 10^−10^	16
GO:0044267	cellular protein metabolic process	BP	2.51 × 10^−8^	147
GO:0005737	cytoplasm	CC	4.47 × 10^−8^	138
GO:0044724	single-organism carbohydrate catabolic process	BP	4.96 × 10^−8^	13
GO:0006096	glycolysis	BP	6.23 × 10^−8^	11
GO:0016052	carbohydrate catabolic process	BP	8.55 × 10^−8^	13
GO:0015935	small ribosomal subunit	CC	1.40 × 10^−7^	9
GO:0019538	protein metabolic process	BP	2.35 × 10^−7^	171
GO:0006091	generation of precursor metabolites and energy	BP	3.55 × 10^−7^	27
GO:0006006	glucose metabolic process	BP	6.83 × 10^−7^	13
GO:0006007	glucose catabolic process	BP	9.27 × 10^−7^	11
GO:0019320	hexose catabolic process	BP	1.17 × 10^−6^	11
**M6 vs. M3**				
GO:0015204	urea transmembrane transporter activity	MF	0.017515	2
GO:0015840	urea transport	BP	0.017515	2
GO:0019755	one-carbon compound transport	BP	0.017515	2
GO:0071918	urea transmembrane transport	BP	0.017515	2

CC, MF, and BP are abbreviations for cellular component, molecular function, and biological process, respectively.

**Table 5:** The most enriched GO terms of target genes of DE-lncRNAs in the M6 vs. M3 comparison.

GO Accession	Description	Term Type	*p*-Value	Gene Count
GO:0015204	urea transmembrane transporter activity	MF	0.047565	2
GO:0015840	urea transport	BP	0.047565	2
GO:0019755	one-carbon compound transport	BP	0.047565	2
GO:0071918	urea transmembrane transport	BP	0.047565	2

**Table 6:** The most enriched KEGG pathways of DE-mRNAs in the M0 vs. M6 comparison.

KEGG Pathway	Rich Factor	*p*-Value	Gene Number
Ribosome	0.211073	4.03 × 10^−12^	61
Biosynthesis of amino acids	0.25	0.000259	19
Glycolysis/gluconeogenesis	0.241935	0.00275	15

**Table 7:** The most significantly enriched KEGG pathways of target genes co-located with DE-lncRNAs.

Comparison	KEGG Pathway	Rich Factor	*p*-Value	Gene Number
**M0 vs. M3**	2-oxocarboxylic acid metabolism	0.176471	0.02762	3
**M0 vs. M6**	Ubiquitin-mediated proteolysis	0.05036	0.025317	7

**Table 8:** Pairs of DE-mRNAs and DE-lncRNAs.

lncRNA id	log2FoldChange	mRNA id	Gene Name	log2FoldChange
**M0 vs. M3**				
TCONS_00074191	12.80220475	ENSCHIG00000007982	*METTL11B*	−3.86509
TCONS_00136308	13.66725117	XLOC_087192	*XLOC_087192*	1.83846
TCONS_00074190	11.40821788	ENSCHIG00000007982	*METTL11B*	−3.86509
**M0 vs. M6**				
TCONS_00135868	−15.24605461	ENSCHIG00000017944	*NRG4*	−1.63975
TCONS_00045045	14.24586981	ENSCHIG00000024367	*NBEA*	1.245331
TCONS_00078361	13.30525325	ENSCHIG00000007982	*METTL11B*	−4.7664
TCONS_00135347	12.15929256	ENSCHIG00000017618	*PDE8A*	−1.74242
TCONS_00214300	−4.18296294	ENSCHIG00000013176	*OR2AP1*	−4.19294
TCONS_00167695	−11.75820204	ENSCHIG00000020411	*A1CF*	−12.2245
TCONS_00045031	13.47115691	ENSCHIG00000024367	*NBEA*	1.245331
TCONS_00142330	−10.67270908	ENSCHIG00000017389	*ATP2B2*	−2.93845
**M6 vs. M3**				
TCONS_00190312	−12.28261621	ENSCHIG00000007731	*NGF*	−4.65694
TCONS_00182938	13.70600879	ENSCHIG00000015813	*GRM5*	9.54066

## Data Availability

The datasets generated and/or analyzed during the current study are available in the data base SRA (Sequence Read Archive) in the NCBI repository, under BioProject ID PRJNA795300.
